# Evaluation of the Concentration of Selected Elements in Patients with Cancer of the Reproductive Organs with Respect to Treatment Stage—Preliminary Study

**DOI:** 10.3390/nu14122368

**Published:** 2022-06-07

**Authors:** Sylwia Wieder-Huszla, Anita Chudecka-Głaz, Aneta Cymbaluk-Płoska, Beata Karakiewicz, Mateusz Bosiacki, Dariusz Chlubek, Anna Jurczak

**Affiliations:** 1 Department of Clinical Nursing, Pomeranian Medical University in Szczecin, 71-210 Szczecin, Poland; sylwia.wieder.huszla@pum.edu.pl (S.W.-H.); anna.jurczak@pum.edu.pl (A.J.); 2 Department of Gynecological Surgery and Gynecological Oncology of Adults and Adolescents, Pomeranian Medical University in Szczecin, 70-111 Szczecin, Poland; aneta.cymbaluk@pum.edu.pl; 3 Department of Social Medicine and Public Health, Pomeranian Medical University in Szczecin, 71-210 Szczecin, Poland; beata.karakiewicz@pum.edu.pl; 4 Department of Functional Diagnostics and Physical Medicine, Pomeranian Medical University in Szczecin, 70-111 Szczecin, Poland; bosiacki.m@pum.edu.pl; 5 Department of Biochemistry and Medical Chemistry, Pomeranian Medical University in Szczecin, 70-111 Szczecin, Poland; dchlubek@pum.edu.pl

**Keywords:** biochemical parameters, ovarian cancer, endometrial cancer, chemotherapy, women’s health

## Abstract

(1) Background: The aim of this study was to assess the concentrations of selected elements in female patients with cancer of the reproductive organs, taking into account the stage of treatment. (2) Methods: The study sample consisted of 51 patients with advanced endometrial cancer and ovarian cancer, undergoing chemotherapy. The median age of the studied patients with endometrial cancer was 66.0 years (IQR: from 60.75 to 70.25), and with ovarian cancer―60.0 years (IQR: from 49.0 to 64.0). Each of the qualified women, after consent to participate in the study, had her blood drawn several times (before surgery, the first course of chemotherapy, the third course of chemotherapy, and the sixth course of chemotherapy) in order to determine serum levels of macro- and micronutrients (Na, Mg, Ca, Zn, P, Cu, Fe, Cd, Ni, and Sr). (3) Results: In the study group of patients with cancer of the reproductive tract, the concentrations of iron (<0.001), magnesium (0.038), sodium (0.014), and nickel (0.037) varied significantly over the course of the study. The analysis showed that the interaction between the stage of chemotherapy and the type of cancer had an effect on the concentrations of magnesium and cadmium (*p* < 0.05). (4) Conclusions: In the studied group of patients with ovarian and endometrial cancer, the applied chemotherapy significantly changed the concentrations of Fe, Na, and Ni, regardless of the type of tumor. Changes in Mg and Cd concentrations resulted from the interaction between the stage of chemotherapy and the type of cancer. The results of serum concentrations of selected elements in women with cancer of the reproductive organs may help understand the physiological changes resulting from the applied chemotherapy.

## 1. Introduction

Neoplastic diseases of the female reproductive organs are a serious health and social problem because the diagnosis of oncological disease, the commencement of therapy, and functioning after the disease change women’s lives completely, often evoking feelings of insecurity, threat, and loss of femininity. These conditions are associated with a variety of both physical and emotional complaints. Oncological treatment (radiotherapy and chemotherapy) entails a number of complications. Consequently, all these aspects affect the quality of women’s lives, both during the treatment and after its completion. The most common cancer of the female reproductive organs is endometrial cancer, which is usually found in women over 50 years of age. The upward trend of this cancer in developed countries, which also include Poland, is confirmed by the epidemiological data collected by WHO and the National Cancer Registry in Poland [1,2]. Another common malignancy is ovarian cancer [3], with approximately 240,000 cases diagnosed annually worldwide and approximately 150,000 women dying as a result [4,5]. The treatment of these genital tract cancers is mostly based on combined therapy, i.e., surgery followed by systemic chemotherapy or radiotherapy. Systemic chemotherapy after appropriate surgical treatment is often the mainstay of combination therapy. First-line chemotherapy for these cancers involves the use of platinum-based regimens in combination with paclitaxel, administered every 21 days in the amount of six courses [6]. Due to the lack of valuable screening tests, often late diagnosis, and poor prognosis, it seems that modifiable risk factors are extremely important preventive elements. In cancers of the reproductive tract, diagnostic tools include clinical history, physical examination, and additional tests. Perhaps monitoring serum levels of trace elements could complement the diagnosis. The knowledge of how to control the serum levels of certain trace elements and their concentrations that change with age, as well as rational supplementation, may help to reduce the risk of certain types of cancer. Although trace elements are essential for life, both their deficiency and excess may cause a number of disorders threatening human health. There are some suspicions that an excess of some micronutrients can also induce pathological processes such as neoplastic proliferation. On the other hand, other studies show that micronutrient deficiencies observed in oncological patients have a negative impact on the course of cancer and the effectiveness of the treatment, as they impair immunocompetence, increase the risk of complications, and affect the physical and mental quality of the patient’s life. The available results support the importance of micronutrients as adjuvants in nutritional therapy and provide evidence that the intake of mineral/multivitamin supplements improves both the quality of life and prognosis of cancer patients [7,8,9,10,11,12,13,14]. At the same time, the oncology community has signaled the existence of justified concerns about the negative impact of dietary supplements on the effectiveness of chemotherapy and radiotherapy [7,8,9,10,11,12,13,14,15,16,17].

When analyzing trace elements, it is worth considering to what extent environmental chemicals increase the risk of hormonal cancers, namely ovarian and endometrial cancer. Available studies show that metals such as cadmium, zinc, copper, iron, nickel, and aluminum mimic the effects of estrogen. It has been proven that exposure to these substances stimulates the formation of malignant neoplasms [18,19]. In patients with ovarian cancer, an increase in the concentration of copper and zinc is observed, which in turn disturbs the reductive-oxidative balance of the system [19]. In the case of iron, disturbances in its metabolism may promote the formation of neoplastic cells [20], and elevated levels of copper contribute to cancer progression [21]. Additionally, the presence of heavy metals and an increase in the content of trace metals in the body, such as cadmium, nickel, and strontium, may lead to abnormal metabolic reactions, determining the course of cancer disease and the effectiveness of the treatment [22,23,24,25,26,27].

## 2. Materials and Methods

The study involved 51 patients treated in the Department of Gynecological Surgery and Gynecological Oncology of Adults and Adolescents, the Pomeranian Medical University in Szczecin. Informed consent was a prerequisite for participation in the study. The study was conducted in accordance with the Declaration of Helsinki, and the protocol was approved by the Bioethical Commission (Approval no. KB-0012/81/18). Patients with advanced ovarian and endometrial cancer undergoing first-line chemotherapy and systemic treatment for recurrence were included in the study. For ovarian cancer, patients with primary cancer underwent surgery followed by chemotherapy, i.e., 6 courses of chemotherapy based on platinum analogs and paclitaxel, or, in the case of incomplete surgical treatment, additionally received 18 administrations of bevacizumab. For recurrent disease, the choice of chemotherapy depended on platinum sensitivity. Patients with endometrial cancer, in advanced cases, were treated with surgery followed by chemotherapy and radiotherapy. Chemotherapy regimens were based on platinum analogs and paclitaxel in the amount of 6 courses/cycles administered at 21-day intervals. Doxorubicin regimens were used in relapse.

### 2.1. Study Design

The research procedure was divided into three parts:

- A structured interview;
- Anthropometric measurements;
- Serum biochemical analysis.

First, participants were asked about basic sociodemographic data (age, place of residence, employment status, education, and marital status) as well as menstruation, family history of cancer, medications taken, and physical activity. Anthropometric measurements were taken on an empty stomach, in light clothes, without shoes, after emptying the urinary bladder using an electronic scale with a height gauge. Based on the obtained data, the body mass index (BMI) was calculated―the range of 18.5–24.9 kg/m^2^ was regarded as normal, overweight was defined as having a BMI in the range of 25.0–29.9 kg/m^2^, and obesity—BMI of 30 kg/m^2^ and more. Following the study protocol, each of the qualified women, after consent to participate in the study, had her blood drawn several times (before surgery, the 1st course of chemotherapy, the 3rd course of chemotherapy, and the 6th course of chemotherapy). Venous blood (maximum 5.5 mL) was collected on an empty stomach (minimum eight hours from the last meal) using the Monovette system in order to determine serum levels of macro- and micronutrients (Na—the norm: 3104–3333 mg/L; Mg—the norm: 20–25 mg/L; Ca—the norm: 89–101 mg/L; Zn—the norm: 0.66–1.1 mg/L; P—the norm: 30–45 mg/L; Cu—the norm: 0.8–1.6 mg/L; Fe—the norm: 0.35–1.5 mg/L; Cd—the norm: 0.0049 mg/L; Ni—the norm: 0.002 mg/L; Sr—the norm: 0.05 mg/L). After obtaining the biological material, the blood was centrifuged. In total, 154 serum samples were obtained for further analysis, i.e., ovarian cancer (*n* = 81) and endometrial cancer (*n* = 73). The separated serum was kept frozen at −80 °C until analysis at the Department of Biochemistry and Medical Chemistry, Pomeranian Medical University in Szczecin.

### 2.2. Determination of Biochemical Parameters

Determinations of biochemical parameters were performed in a certified laboratory of the Pomeranian Medical University in Szczecin using commercial, standardized methods.

### 2.3. Analysis of Serum Element Concentrations

All samples were transferred into 1.5 mL microtubes and stored at −80 °C until processed.

Samples were analyzed using inductively coupled plasma optical emission spectrometry (ICP-OES, ICAP 7400 Duo, Thermo Fisher Scientific, Waltham, MA, USA) equipped with a concentric nebulizer and a cyclonic spray chamber to determine their Ca, Zn, Cu, Fe, Na, Sr, P, Mg, Cd, and Ni content. Analysis was performed in radial and axial mode. The samples were thawed at room temperature and digested using the microwave digestion system MARS 5, CEM. The volume of the sample given to the research was 0.75 mL.

The samples were transferred to clean polypropylene tubes; 4 mL of 65% HNO_3_ (Suprapur, Merck, Darmstadt, Germany) was added to each vial, and each sample was allowed 30 min pre-reaction time in the clean hood. After completion of the pre-reaction time, 1 mL of non-stabilized 30% H_2_O_2_ solution (Suprapur, Merck, Darmstadt, Germany) was added to each vial. Once the addition of all reagents was complete, the samples were placed in special Teflon vessels and heated in microwaved digestion system for 35 min at 180 °C (15 min ramp to 180 °C and maintained at 180 °C for 20 min). At the end of digestion, all samples were removed from the microwave and allowed to cool to room temperature. In the clean hood, the samples were transferred to acid-washed 15 mL polypropylene sample tubes. A further 5-fold dilution was performed prior to ICP-OES measurement. The volume of 2 mL was taken from each digest. The samples were spiked with an internal standard to provide a final concentration of 0.5 mg/L Ytrium, 1 mL of 1% Triton (Triton X-100, Sigma, Darmstadt, Germany), and diluted to the final volume of 10 mL with 0.075% nitric acid (Suprapur, Merck, Darmstadt, Germany). The samples were stored in a monitored refrigerator at a nominal temperature of 8 °C until analysis.

Blank samples were prepared by adding concentrated nitric acid to tubes without sample and subsequently diluted in the same manner as described above.

Multi-element calibration standards (ICP multi-element standard solution IV, Merck and Phosphorus ICP Standard (AccuStandard, Inc., New Haven, CT, USA) were prepared with different concentrations of inorganic elements in the same manner as blanks and samples.

Deionized water (Direct Q UV, Millipore, Darmstadt, Germany, approximately 18.0 MΩ) was used to prepare all solutions. The reference material samples (NIST SRM 8414 Bovine Muscle) were prepared in the same manner as the samples. The wavelengths (nm) were as follows: Ca 315.887, Zn 206.200, Cu 224.700, Fe 259.94, Na 589.592, Sr 421.552, P 178.284, Mg 280.270, Cd 228.802, and Ni 221.647.

### 2.4. Statistical Analysis

All analyses in the present study were performed in MedCalc (ver.20.007, Ostend, Belgium) and R software (https://www.r-project.org, accessed on 16 February 2022). First, for clarity, raw measurements were presented separately to give the reader an overview of the data. In the case of values below the limit of detection (in particular for Cd, Ni, and Sr), the lowest measurable value was used in a dataset. This approach, however, was only used to present basic descriptive statistics and for correlation analyses by Spearman’s method.

For further calculations, the data were log-transformed to conform to normality. We performed the repeated measures ANOVA with two corrections based on the estimates of sphericity by Greenhouse and Geisser or Huynh and Feldt as appropriate. Next, we utilized linear mixed models followed by the maximum likelihood ratio test to deal with missing data and to search for the interaction between the group (type of cancer) and the stage of therapy. Where necessary, Satterthwaite’s method was used to obtain the *p* values. The dependent variable was the log concentration of a particular tested element, while the independent ones were the stage of therapy and the type of cancer (both fixed). The Patient ID was the only random variable. The statistical significance was set as *p* < 0.05.

## 3. Results

### 3.1. Characteristics of the Participants

The study involved 51 women diagnosed with ovarian (*n* = 26, 52%) or endometrial (*n* = 24, 48%) cancer. The age of patients suffering from endometrial cancer was significantly higher compared to that of women with ovarian cancer (median: 66.0 years old; IQR: from 60.750 to 70.25 vs. median: 60.0 years; IQR: from 49.0 to 64.0, *p* < 0.0061). The women in both groups had their first period at the age of 14 years (*p* = 0.4546). The BMI of the participants remained stable over time (*p* = 0.864). However, in terms of weight status over time, we noticed that obesity was more prevalent in women with endometrial cancer. Other variables are shown in Table 1.

### 3.2. The Concentrations of the Tested Elements at the Surgery and during Chemotherapy

The basic descriptive statistics of the tested elements are shown in Table 2. In patients with ovarian cancer, an increase in Fe concentration was observed in the subsequent stages of treatment, as the mean level before the procedure oscillated around 1.1620 (SD = 0.6060), reaching higher values before the third and sixth cycle of chemotherapy, 2.2090 ± 1.1307 and 2.2000 ± 0.9382, respectively. The increase in Fe concentration in patients with uterine cancer was also evident with time; before surgery (1.175 ± 0.6189) vs. the third cycle (1.829 ± 0.8677) and the sixth cycle (1.987 ± 0.8455). The decrease in Mg concentration was observed especially in patients with uterine cancer: the initial mean value was 26.831 (SD =2.5907), whilst before the sixth cycle of chemotherapy, it decreased to 22.082 (SD =3.4344). Such a decrease was also noticed in patients with ovarian cancer but to a smaller extent. Electrolyte concentration alterations—especially in regard to lowering levels of Na and P along with the treatment—were demonstrated in both groups of patients. In turn, Ni concentrations increased along with the treatment in both groups. For further analysis, the data were log-transformed. We noticed that the concentrations of Fe (<0.001), Mg (0.038), Na (0.014), and Ni (0.037) differed significantly by the stage of therapy. The results are presented in Table 3.

### 3.3. The Concentrations of the Tested Elements by the Stage of Therapy and the Type of Cancer

**Figure 1:**
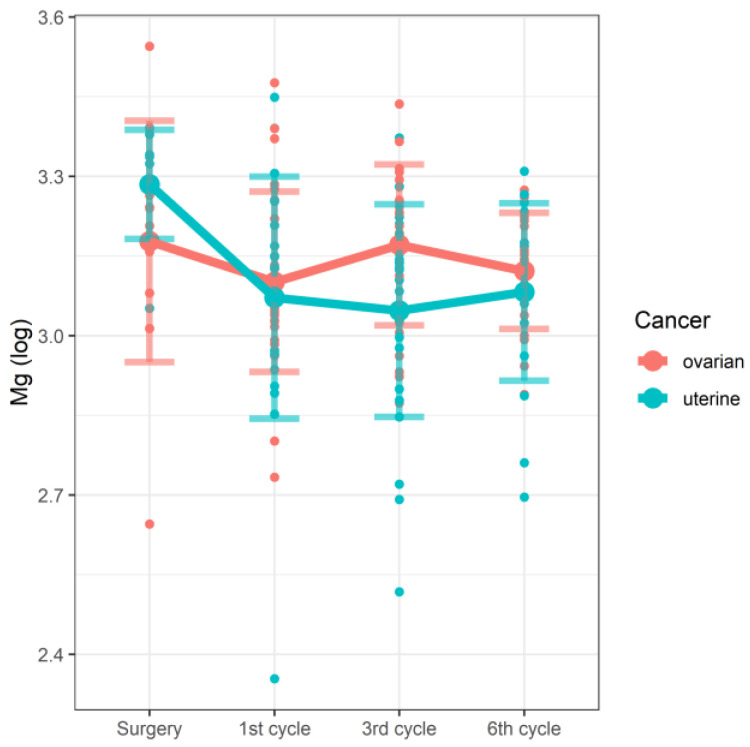
Changes in Mg concentrations (log) by the stage of therapy and the type of cancer.

**Figure 2:**
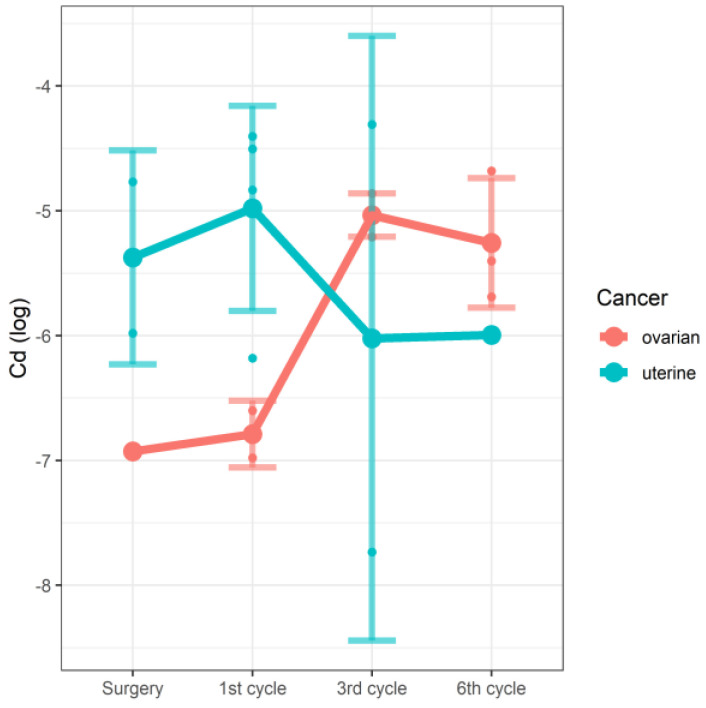
Changes in Cd concentrations (log) by the stage of therapy and the type of cancer.

In the linear mixed model, we were able to demonstrate the effect of the interaction between the stage of therapy and the type of cancer on the concentrations of Mg (Figure 1) and Cd (Figure 2). In the case of both tested elements, the changes in concentrations observed along the study time points were significantly associated with cancer type. In the studied group of patients with cancer of the reproductive tract, serum magnesium (Mg) concentrations correlated with the cycle of chemotherapy and the type of cancer. However, due to the very limited number of raw data concerning Cd, these results should be treated with some caution. The details are shown in Table 4.

### 3.4. The Correlations between the Concentrations of the Tested Elements and the Stage of Therapy

In the following analysis, we treated the stage of therapy—being interval variables—as quantitative data. We demonstrated that the concentration of Fe was positively correlated with the stage of therapy irrespective of the type of cancer (Table 5).

## 4. Discussion

Monitoring serum levels of elements can be a helpful diagnostic tool in the prevention and early detection of many neoplastic diseases [28]. Thus, the analysis of the concentrations of individual bioelements in the body may help objectivize the results of various studies. Many authors confirm the correlation between selected elements and specific disease states at certain stages of a woman’s life. According to the current state of knowledge, macro- and microelements regulate the development and functioning of immune system cells. In turn, modulation of the immune response by these components may be an effective method in reducing the risk of disease and/or treating certain diseases, but their role in immune regulation and in pathologies of the immune system has not yet been fully understood [28]. It is very important to maintain the correct proportions between elements because both their deficiency and excess may lead to physiological disorders [29].

Numerous studies confirm that oxygen free radicals and associated inflammation play an important role in the multifactorial pathogenesis of ovarian cancer, in addition to genetic, epigenetic, and hormonal factors [30]. According to Roychoudhury’s team, copper, by forming complexes with gonadoliberin, increases the secretion of follicle-stimulating hormone (FSH) and luteinizing hormone (LH), which may contribute to the development of ovarian cancer [31]. Gupte et al. demonstrated elevated copper levels in, among others, patients with ovarian and cervical cancer. Higher copper levels were directly related to cancer progression. According to the researchers, this element is involved in the angiogenesis of the tumor growth process [21]. Our study did not show any differences in serum copper concentrations between the studied patients, regardless of the diagnosed cancer (ovarian cancer vs. uterine cancer) and the stage of oncological treatment. On the other hand, the study by Atakul et al. demonstrated lower blood Cu and Zn concentrations as well as a lower Cu/Zn ratio in women with endometrial cancer compared to the healthy group. The results also confirm the correlation of Cu and Zn levels with age [32]. Rzymski et al. observed elevated cadmium (Cd) levels in neoplastic endometrial tissues, as well as altered Cu and Zn values [33]. Our results showed that cadmium levels were significantly affected by tumor type and the cycle of chemotherapy administered. In ovarian cancer, Cd levels increased in the third cycle of chemotherapy and then slightly decreased in the sixth cycle. In endometrial cancer, on the other hand, the concentration slightly increased in the first cycle of chemotherapy, then decreased in the third cycle, and remained at a similar level in the sixth cycle.

The influence of low magnesium concentrations on the treatment outcomes, prognosis, and survival of patients with advanced ovarian cancer undergoing chemotherapy is not fully understood [34]. The study by Castiglioni and Maier confirmed that serum magnesium levels in patients with precancerous lesions are decreased regardless of the therapy administered and correlate with the stage of cancer [35]. The results obtained by Yao et al. [36] showed that the concentration of magnesium decreased at the end of the first week of radiotherapy and after the introduction of chemotherapeutic agents that cause magnesium loss, such as cisplatin [37,38,39,40,41,42,43,44]. Among the side effects of chemotherapy, nephrotoxic implications and electrolyte disturbances, especially hypomagnesemia, are most commonly described [45]. In our research, the concentration of Mg correlated with the stage of treatment/cycle of chemotherapy. In the group of patients with endometrial cancer, the level of magnesium decreased in the first and third cycle of chemotherapy; then, it slightly increased during the sixth course. In patients with ovarian cancer, on the other hand, Mg levels decreased in the first and sixth cycles, while an increase was obtained in the third cycle.

Undoubtedly, the process of carcinogenesis may be influenced by changes in iron metabolism. In women, the level of this element depends on the period of life. In menstruating women, anemia and iron deficiency are associated with elevated levels of vascular endothelial growth factor (VEGF), which contributes to tumor angiogenesis. High concentrations of estrogens also play a negative role because free radicals produced in the metabolic cycle of estradiol contribute to the release of iron from ferritin, which, when unbound to protein, contributes to DNA damage. After menopause, the cessation of menstruation minimizes the loss of iron; however, overloading the body with this element may enhance the process of carcinogenesis as a result of accelerated production of reactive oxygen species (ROS) [46,47]. Another important and still unclear issue regarding Fe is the effect of cisplatin (CDDP)-based chemotherapy on the systemic balance of this metal. Yamashita and Nakamura’s teams confirm an increase in serum Fe levels in patients undergoing cisplatin-based chemotherapy [48,49]. Changes in iron levels after the first day of high doses of cisplatin in cancer patients were indicated by Sartori et al. [50], who observed in these patients hypersideremia developing within 24 h [51]. A significant increase in iron levels found in patients with ovarian cancer at the end of a 5-day cisplatin course was shown in the study by Poller et al. [52]. We also observed a significant effect of the applied chemotherapy on the concentration of iron. An increase in Fe levels was observed in both ovarian and endometrial cancer patients during the third and sixth cycles of chemotherapy.

Electrolyte balance plays a vital role in the functioning of cells and organs. Patients with cancer are particularly vulnerable to electrolyte disturbances. The study by Omoyajowo et al. carried out to estimate the level of electrolytes in a group of patients with uterine cancer showed a significant increase in the concentrations of PO_4_^3−^ (*p* < 0.05) and Na^+^ (*p* < 0.0001). There was no significant difference in the concentration of K^+^ in this study (*p* > 0.05). The authors argue that electrolyte imbalance determines the prognosis, diagnosis, and management of uterine cancer [53]. In our study, sodium levels in patients in both groups (ovarian cancer vs. endometrial cancer), compared to the results obtained before surgery, decreased significantly with subsequent cycles of chemotherapy.

Malignant neoplasms of female genital organs are diseases with a serious prognosis, taking a heavy toll on the body and its performance. While treating the underlying disease, i.e., cancer, it is also necessary to fight the side effects of cancer therapy. Further research on trace element monitoring may determine the effectiveness of cancer treatment and thus have a positive impact on treatment results and extend the survival time of patients.

### Limitations

In our study, we did not perform a detailed analysis regarding the use of vitamin and mineral supplements by the studied patients. Our goal was to make a preliminary assessment of the concentrations of selected elements. We were afraid that the participants might hide the truth or provide unreliable information if they were asked more detailed questions about the vitamin preparations they were taking.

## 5. Conclusions

(1) In the studied group of patients with ovarian and endometrial cancer, the applied chemotherapy significantly changed the concentrations of Fe, Na, and Ni regardless of the type of tumor.

(2) Changes in Mg and Cd concentrations result from the interaction of the duration of chemotherapy and the type of tumor.

(3) The results of serum concentrations of selected elements in women with cancer of the reproductive organs may help understand the physiological changes resulting from the applied chemotherapy.

## Figures and Tables

**Table 1:** Sociodemographic data.

**Variable**	**Level of Education**				*p*
Cancer Type	Primary	Vocational	Secondary	Higher	
Ovarian cancer	3	5	13	5	0.59
Endometrial cancer	1	8	11	4	
	Marital status				*p*
Cancer type	Unmarried	Married	Widowed	Divorced	
Ovarian cancer	3	10	11	2	0.49
Endometrial cancer	2	5	15	2	
	Employment status				*p*
Cancer type	Employed	Unemployed	Sickness pension	Pension	
Ovarian cancer	9	5	11	1	0.79
Endometrial cancer	6	4	12	2	
	Place of residence				*p*
Cancer type	Village	City with a population under 10,000	City with a population of 10,000–100,000	City with a population of >100,000	
Ovarian cancer	4	4	9	9	0.48
Endometrial cancer	4	8	6	6	
	BMI				*p*
Cancer type	Normal weight	Obese	Overweight	Underweight	
Ovarian cancer	30	19	28	3	0.0032
Endometrial cancer	17	38	15	4	

*p*—significance level.

**Table 2:** Descriptive statistics of the tested elements by the type of cancer and the stage of therapy.

Variable	Ovarian Cancer															
	Surgery (*n* = 11)				1st Cycle (*n* = 26)				3rd Cycle (*n* = 24)				6th Cycle (*n* = 20)			
	M	Me	SD	25–75 P	M	Me	SD	25–75 P	M	Me	SD	25–75 P	M	Me	SD	25–75 P
Ca	122.2130	118.9650	15.4327	107.826–130.972	119.8880	115.6750	18.0889	107.096–126.459	132.8760	131.8890	24.1160	116.873–153.754	125.7940	120.3780	19.4164	113.917–144.401
Cd	0.0004	0.0003	0.0002	0.000330–0.000330	0.0004	0.0003	0.0002	0.000330–0.000330	0.0011	0.0003	0.0021	0.000330–0.000330	0.0011	0.0003	0.0022	0.000330–0.000330
Cu	1.7360	1.8380	0.4284	1.434–2.106	1.5610	1.4870	0.3109	1.383–1.738	1.4860	1.4450	0.4025	1.195–1.668	1.5660	1.5300	0.2756	1.402–1.679
Fe	1.1620	1.1000	0.6060	0.758–1.154	1.2270	1.1400	0.6332	0.816–1.528	2.2090	1.8170	1.1307	1.499–2.649	2.2000	1.9500	0.9382	1.437–2.967
Mg	24.5280	24.7090	5.1852	22.212–26.020	22.5440	21.4910	3.9238	20.437–25.036	24.0880	24.9970	3.5235	20.900–26.263	22.8180	22.9270	2.4318	21.280–25.038
Na	3918.0500	3996.5980	311.9501	3657.605–4102.884	3368.0200	3463.8950	376.2940	3166.725–3587.460	3617.3110	3720.3850	371.1486	3335.260–3913.682	3581.6050	3629.4930	338.6305	3341.971–3847.588
Ni 2	0.0015	0.0008	0.0020	0.000770–0.000770	0.0029	0.0008	0.0050	0.000770–0.000770	0.0064	0.0008	0.0072	0.000770–0.0121	0.0043	0.0008	0.0061	0.000770–0.00732
P	369.4810	401.3790	149.9710	214.098–451.331	228.2620	183.8610	111.5522	162.129–229.317	246.4140	218.7890	115.5608	184.970–259.033	278.5980	209.3050	118.9908	193.992–398.847
Sr	0.0423	0.0291	0.0433	0.00972–0.0638	0.0542	0.0575	0.0318	0.0322–0.0776	0.0588	0.0597	0.0316	0.0385–0.0852	0.0488	0.0491	0.0185	0.0324–0.0610
Zn	2.3530	1.6960	1.3231	1.177–3.654	2.4600	2.0950	1.9281	1.203–2.677	2.4600	2.3950	1.1458	1.412–3.202	2.4240	2.0430	1.1056	1.552–3.330
Variable	Endometrial cancer															
	Surgery (*n* = 10)				1st cycle (*n* = 21)				3rd cycle (*n* = 24)				6th cycle (*n* = 18)			
Ca	121.907	119.281	12.4636	115.115–124.657	123.221	120.833	16.454	115.850–128.818	121.806	113.47	28.4485	96.114–136.605	121.277	122.043	21.9249	106.810–136.223
Cd	0.00137	0.00033	0.0026	0.000330–0.000330	0.001850	0.00033	0.003668	0.000330–0.000330	0.000881	0.00033	0.002677	0.000330–0.000330	0.00045	0.00033	0.00051	0.000330–0.000330
Cu	1.421	1.327	0.3331	1.125–1.691	1.556	1.548	0.2484	1.375–1.699	1.488	1.486	0.2888	1.252–1.651	1.53	1.511	0.3519	1.245–1.621
Fe	1.175	1.215	0.6189	0.835–1.353	1.222	1.228	0.524	0.926–1.334	1.829	1.683	0.8677	1.322–2.291	1.987	1.98	0.8455	1.327–2.383
Mg	26.831	27.291	2.5907	26.580–28.243	22.072	22.054	4.5432	19.263–24.022	21.438	22.755	3.9275	18.905–23.472	22.082	22.271	3.4344	20.567–23.940
Na	3746.173	3837.134	393.5432	3629.755–3956.274	3372.38	3441.793	339.4302	3135.612–3678.553	3384.109	3405.028	462.3386	3031.447–3813.382	3560.183	3714.223	549.641	3091.267–3847.698
Ni 2	0.00077	0.00077	0	0.000770–0.000770	0.00352	0.00077	0.005297	0.000770–0.00219	0.00335	0.00077	0.004522	0.000770–0.00403	0.00302	0.00077	0.004937	0.000770–0.00357
P	316.265	244.567	162.7694	189.481–510.812	195.062	197.239	49.8238	158.817–215.579	247.982	199.012	116.664	169.353–304.745	232.496	202.774	106.6331	173.238–218.308
Sr	0.0265	0.0138	0.03619	0.00341–0.0393	0.0646	0.0666	0.02715	0.0455–0.0798	0.0578	0.0518	0.03343	0.0342–0.0816	0.0561	0.0455	0.03688	0.0278–0.0750
Zn	2.797	2.404	1.5473	1.937–2.888	2.177	2.175	0.8629	1.539–2.414	2.368	2.126	1.2732	1.447–2.939	2.635	2.174	1.5041	1.478–3.116

M—mean; Me—median; SD—standard deviation; P—percentile.

**Table 3:** The relationship between the levels of the tested elements, the stage of therapy, and the type of cancer.

Stage of Therapy	Element	Ovarian Cancer				Endometrial Cancer				*	**
		Min	M	Me	25–75 P	Min	M	Me	25–75 P		
Surgery	Ca	4.658	4.799	4.779	4.680–4.875	4.668	4.799	4.781	4.746–4.826	0.267	0.135
1st cycle		4.55	4.776	4.751	4.674–4.840	4.549	4.806	4.794	4.752–4.858		
3rd cycle		4.477	4.873	4.882	4.761–5.035	4.498	4.778	4.731	4.566–4.917		
6th cycle		4.556	4.824	4.791	4.735–4.973	4.433	4.783	4.804	4.671–4.914		
Surgery	Cd	−6.928	−6.928	−6.928	−6.928–−6.928	−5.98	−5.374	−5.374	−5.980–−4.768	0.47	0.96
1st cycle		−6.978	−6.789	−6.789	−6.978–−6.600	−6.18	−4.981	−4.67	−5.507–−4.454		
3rd cycle		−5.209	−5.035	−5.033	−5.165–−4.905	−7.733	−6.021	−6.021	−7.733–−4.309		
6th cycle		−5.689	−5.257	−5.402	−5.617–−4.861	−5.994	−5.994	−5.994	−5.994–−5.994		
Surgery	Cu	0.0452	0.521	0.609	0.360–0.743	0.035	0.328	0.283	0.118–0.525	0.557	0.892
1st cycle		0.0881	0.427	0.397	0.325–0.553	0.142	0.43	0.437	0.318–0.530		
3rd cycle		0.0452	0.367	0.368	0.178–0.512	0.0719	0.38	0.396	0.224–0.501		
6th cycle		0.158	0.435	0.425	0.338–0.518	0.0512	0.402	0.412	0.219–0.483		
Surgery	Fe	−0.633	0.0454	0.0949	−0.278–0.143	−1.06	0.0335	0.193	−0.180–0.302	0.595	<0.001
1st cycle		−1.124	0.0747	0.131	−0.204–0.424	−1.592	0.0959	0.205	−0.0769–0.288		
3rd cycle		−0.393	0.687	0.596	0.405–0.974	−0.537	0.49	0.52	0.276–0.827		
6th cycle		0.13	0.705	0.661	0.363–1.088	−0.284	0.597	0.683	0.283–0.868		
Surgery	Mg	2.645	3.178	3.207	3.100–3.259	3.052	3.285	3.306	3.280–3.341	0.748	0.038
1st cycle		2.734	3.101	3.068	3.017–3.220	2.354	3.072	3.093	2.958–3.179		
3rd cycle		2.873	3.171	3.219	3.040–3.268	2.518	3.047	3.125	2.939–3.156		
6th cycle		2.89	3.122	3.132	3.058–3.220	2.696	3.082	3.103	3.024–3.176		
Surgery	Na	8.159	8.271	8.293	8.205–8.319	7.94	8.223	8.252	8.197–8.283	0.187	0.014
1st cycle		7.832	8.116	8.15	8.060–8.185	7.879	8.118	8.144	8.051–8.210		
3rd cycle		7.92	8.188	8.222	8.112–8.272	7.813	8.118	8.133	8.017–8.246		
6th cycle		7.989	8.179	8.197	8.114–8.255	7.895	8.166	8.22	8.036–8.255		
Surgery	Ni	−6.237	−5.57	−5.57	−6.237–−4.904	-	-	-	-	0.069	0.037
1st cycle		−6.207	−4.85	−4.636	−5.456–−4.225	−5.042	−4.44	−4.369	−4.573–−4.212		
3rd cycle		−5.362	−4.433	−4.402	−4.653–−4.218	−6.768	−5.155	−4.913	−5.766–−4.384		
6th cycle		−5.16	−4.464	−4.453	−4.722–−4.082	−5.634	−4.916	−5.139	−5.352–−4.457		
Surgery	P	5.158	5.829	5.995	5.366–6.112	4.972	5.639	5.493	5.244–6.236	0.053	0.201
1st cycle		4.977	5.347	5.214	5.088–5.435	4.739	5.245	5.284	5.067–5.373		
3rd cycle		4.853	5.431	5.388	5.220–5.555	4.676	5.421	5.293	5.131–5.718		
6th cycle		4.977	5.549	5.344	5.268–5.988	5.029	5.376	5.312	5.155–5.386		
Surgery	Sr	−5.137	−3.296	−3.372	−3.781–−2.613	−5.681	−3.945	−3.824	−4.851–−3.202	0.639	0.07
1st cycle		−5.165	−3.119	−2.853	−3.418–−2.543	−4.004	−2.851	−2.71	−3.094–−2.529		
3rd cycle		−4.985	−2.968	−2.81	−3.209–−2.440	−6.26	−3.018	−2.828	−3.350–−2.497		
6th cycle		−3.777	−3.088	−3.015	−3.430–−2.797	−3.931	−3.065	−3.092	−3.581–−2.590		
Surgery	Zn	−0.317	0.687	0.528	0.162–1.295	0.0947	0.908	0.877	0.661–1.060	0.436	0.637
1st cycle		−0.0352	0.729	0.739	0.185–0.985	0.223	0.715	0.777	0.431–0.881		
3rd cycle		−0.114	0.788	0.873	0.341–1.164	−0.216	0.739	0.754	0.364–1.078		
6th cycle		−0.0358	0.79	0.714	0.440–1.199	0.205	0.84	0.776	0.391–1.136		

M—mean; SD—standard deviation; Me—median; Min—minimum; P—percentile; * type of cancer; ** stage of treatment.

**Table 4:** The concentrations of Mg and Cd depending on the stage of therapy and the type of cancer.

Stage of Therapy	Estimate	Standard Error	df	t	*p*
Mg					
Ovarian surgery	3.19190	0.04811	153.85117	66.347	<2 × 10^−16^
Ovarian 1st cycle	−0.09045	0.05128	117.94225	−1.764	0.0803
Ovarian 3rd cycle	−0.02175	0.05244	120.95496	−0.415	0.6790
Ovarian 6th cycle	−0.07223	0.05482	124.49789	−1.317	0.1901
Endometrial surgery	0.10228	0.06969	153.88577	1.468	0.1442
Endometrial 1st cycle	−0.13273	0.07560	121.30789	−1.756	0.0817
Endometrial 3rd cycle	−0.22711	0.07552	122.42949	−3.007	0.0032
Endometrial 6th cycle	−0.14127	0.07881	123.15295	−1.792	0.0755
Cd					
Ovarian surgery	−6.9280	0.7184	18.0000	−9.644	1.56 × 10^−8^
Ovarian 1st cycle	0.1386	0.8798	18.0000	0.158	0.8766
Ovarian 3rd cycle	1.32	0.8295	18.0000	2.282	0.0348
Ovarian 6th cycle	1.04	0.8295	18.0000	2.014	0.0592
Endometrial surgery	1,43	0.8798	18.0000	1.767	0.0942
Endometrial 1st cycle	0.2543	1.0775	18.0000	0.236	0.8161
Endometrial 3rd cycle	−2.5407	1.0973	18.0000	−2.315	0.0326
Endometrial 6th cycle	−2.2912	1.92	18.0000	−1.895	0.0743

*p*—significance level; df—degrees of freedom; t—distribution.

**Table 5:** The correlations between the concentrations of the tested elements and the stage of therapy.

Element	Whole Group (*N* = 154)		Ovarian Cancer (*n* = 81)	Endometrial Cancer (*n* = 73)
Ca	Correlation coefficient	0.045	0.137	−0.039
	*p*	0.581	0.223	0.7407
Cd	Correlation coefficient	−0.039	0.1	−0.182
	*p*	0.6307	0.3725	0.1225
Cu	Correlation coefficient	−0.051	−0.107	−0.006
	*p*	0.5294	0.3397	0.96
Fe	Correlation coefficient	0.483	0.529	0.426
	*p*	<0.0001	<0.0001	0.0002
Mg	Correlation coefficient	−0.14	−0.027	−0.261
	*p*	0.0826	0.8143	0.0255
Na	Correlation coefficient	−0.037	−0.032	−0.041
	*p*	0.6528	0.7789	0.7279
Ni 2	Correlation coefficient	0.153	0.157	0.154
	*p*	0.059	0.1612	0.1934
P	Correlation coefficient	−0.02	0.004	−0.032
	*p*	0.8017	0.974	0.7898
Sr	Correlation coefficient	0.086	0.057	0.126
	*p*	0.2906	0.6106	0.2894
Zn	Correlation coefficient	0.029	0.07	−0.023
	*p*	0.7235	0.5322	0.8468

*p*—significance level.

## Data Availability

The data presented in this study are available on request from the first author.
